# Pattern of Use of Electronic Health Record (EHR) among the Chronically Ill: A Health Information National Trend Survey (HINTS) Analysis

**DOI:** 10.3390/ijerph18147254

**Published:** 2021-07-07

**Authors:** Rose Calixte, Sumaiya Islam, Zainab Toteh Osakwe, Argelis Rivera, Marlene Camacho-Rivera

**Affiliations:** 1 Department of Epidemiology and Biostatistics, SUNY Downstate Health Sciences University, Brooklyn, NY 11203, USA; 2 CUNY School of Medicine, City College of New York, New York, NY 10031, USA; sislam011@csom.cuny.edu; 3 College of Nursing and Public Health, Adelphi University, Garden City, NY 11530, USA; zosakwe@adelphi.edu; 4 Department of Medicine, Mount Sinai Icahn School of Medicine, New York, NY 10011, USA; argelis.rivera@mountsinai.org; 5 Department of Community Health Sciences, SUNY Downstate Health Sciences University, Brooklyn, NY 11203, USA; marlene.camacho-rivera@downstate.edu

**Keywords:** chronic conditions, patient portal, health communication

## Abstract

Effective patient–provider communication is a cornerstone of patient-centered care. Patient portals provide an effective method for secure communication between patients or their proxies and their health care providers. With greater acceptability of patient portals in private practices, patients have a unique opportunity to manage their health care needs. However, studies have shown that less than 50% of patients reported accessing the electronic health record (EHR) in a 12-month period. We used HINTS 5 cycle 1 and cycle 2 to assess disparities among US residents 18 and older with any chronic condition regarding the use of EHR for secure direct messaging with providers, to request refills, to make clinical decisions, or to share medical records with another provider. The results indicate that respondents with multimorbidity are more likely to share their medical records with other providers. However, respondents who are 75 and older are less likely to share their medical records with another provider. Additionally, respondents who are 65 and older are less likely to use the EHR for secure direct messaging with their provider. Additional health care strategies and provider communication should be developed to encourage older patients with chronic conditions to leverage the use of patient portals for effective disease management.

## 1. Introduction

Patient–physician communication, particularly patients’ satisfaction with physicians’ communication approaches, is important for better outcomes in patient-centered health care organizations [1,2]. Finding effective ways of maintaining communication between health care providers and patients outside of the health care organization is important for disease management and care coordination [1,2]. With legislation mandating the meaningful use of electronic health records (EHR), by 2015, 98% of hospitals and 78% of private practices in the United States offered patient portals [3,4]. Patient portals also provide effective communication tools with the potential to increase patient engagement in self-care management. Patients can also use patient portals for prescription renewal, appointment management, checking lab results, and messaging their providers [5]. Therefore, the use of patient portals is an increasingly common approach in patient-centered care practices [1,2].

With the aging of the US population, certain diseases are becoming more prevalent [6]. Additionally, with people living longer, the prevalence of multimorbidity is also increasing [7,8,9,10,11,12]. Patients with multiple chronic conditions usually require a team of specialists for managing their conditions [13,14]. Maintenance of their treatment plans requires effective communication with patients and coordination of care among their team of providers to achieve better outcomes [15]. Prior research has shown that access to patient portals resulted in improved diabetes-related outcomes and adherence to hypertensive medication [16,17]. Additionally, a recent study of patients with chronic kidney disease (CKD) found that the use of EHR improves patient-centered outcomes such as CKD-specific knowledge, while reducing CKD-related stress [5]. The ongoing COVID-19 pandemic has accelerated the use of technologies such as patient portals for health care delivery [18,19]. Despite the increasing reliance on the use of technology for both preventive and follow-up care, some patients lack resources to engage effectively in telehealth [18].

Despite the proliferation of health-related internet use (HRIU) and the widespread use of the internet by the general population, disparities still exist in terms of access and use of internet for disease management [20,21,22,23,24,25]. Recent studies suggest that despite the increasing trend in the use of patient portals, the acceptability of patient portals in the general population remains unusually low [18]. Additionally, sociodemographic disparities exist in assessing patient portals as a tool for disease management and communication with providers [26,27,28]. A recent study on disparities in health-related internet content that focused on the noninstitutionalized population of the US assessed health information seeking behavior in three domains relevant to health communication (health care, health information-seeking, and user-generated content/sharing). The study indicated age, gender, race/ethnicity, education, and income related disparities across multiple domains of health communication [29].

Several studies have examined potential explanations for low EHR engagement, and HRIU more broadly, within racial and ethnic minority populations. A recent study which examined disparities in trust in sources of cancer-related health information among Hispanics in the US observed that older Hispanics had higher odds of trusting cancer information from a religious organization compared to younger Hispanics [30]. Another study of men with chronic conditions developed an eHealth usage score using seven domains of eHealth communication, which included EHR use [31]. The eHealth questions used to create the score asked whether respondents had done the following: used a computer, smartphone or other electronic means to (1) look for health information or medical information for yourself, (2) look for health or medical information for someone else, (3) buy medicine or vitamins online, (4) look for assistance for the care you provided someone else, (5) use email or the internet to communicate with a doctor or doctor’s office, (6) track health care costs/changes, or (7) look up medical tests. This study identified disparities in eHealth usage across social and demographic characteristics. Particularly, education and income were positively correlated with eHealth score, with participants with higher levels of education and those with higher incomes having increased scores for eHealth usage. However, the same study observed that age and Hispanic ethnicity were negatively correlated with eHealth score, such that older patients had lower scores of eHealth usage, and Hispanics also had lower scores of eHealth usage [31]. Low eHealth usage among individuals of Hispanic ethnicity may be due to low English proficiency and lower levels of health literacy [32,33]. Among older individuals, lower levels of health literacy and technological skill have been found to be associated with lower eHealth usage [34,35]. A previous study examining associations between health literacy and health information seeking found that participants with chronic conditions were more likely to be engaged in health information seeking and higher instances of seeking care based on information found on the Web. Additionally, participants with chronic conditions had higher eHealth literacy scores compared to participants without chronic conditions [36]. When compared to patients without chronic health conditions, patients with a history of chronic conditions reported frequent use of patient portals for different aspects of health care delivery such as checking lab reports, messaging their doctors, and setting up appointments [36]. However, this study failed to adjust for social and demographic factors that are associated with both chronic disease status and health information seeking behaviors. While many studies have looked at disparities in the usage of patient portals in patients with specific chronic conditions, no studies thus far have looked at disparities in the usage of patient portals in only people with chronic conditions in a nationally-representative sample of the noninstitutionalized US population. As EHR usage has been associated with significant improvements in patient self-management of chronic diseases, as well as improved quality of care given by providers, understanding disparities in EHR use may provide important insights into health care disparities among adults living with chronic health conditions in the US [37].

## 2. Materials and Methods

The National Cancer Institute’s Health Information National Trends Survey (HINTS) is a publicly available national representative survey of the noninstitutionalized adult population that collects data about Americans’ use of cancer related information. Data for this study came from the HINTS 5 cycle 1 (*N* = 3335), collected from January 2017 to May 2017, and HINTS 5 cycle 2 (*N* = 3504), collected from January 2018 to May 2018. The sampling design for the HINTS survey has been described extensively [22,38]. The response rate was 32.4% for HINTS 5 cycle 1 and 32.985% for HINTS 5 cycle 2.

The goal of this study was to assess differences in EHR usage among respondents with chronic diseases conditions. Using self-reported data, the study was restricted to respondents with any of the following conditions: diabetes, hypertension, lung disease, heart conditions, depression, cancer, and arthritis. The final analytic sample was further restricted to respondents who reported accessing their online medical record at least once in the past 12 months for various reasons (*N* = 736 and 816 respectively) for a total sample size of 1552. The outcomes of EHR usage were assessed using 4 HINTS questions relating to the purposes of accessing the online medical record to (1) securely message their health care provider, (2) request a refill of medications, (3) make a decision on how to treat illness or condition, and (4) securely share it with another provider.

Primary predictors of interest were gender, race/ethnicity, age, education, income, multimorbidity, and nativity status. The covariates of interest were smoking status, employment status, regular access to a health care provider, insurance status, general health status, and family history of cancer. To measure the change in EHR use across the two HINTS releases, we used a dummy-coded variable to represent the survey year.

We used multivariable regression models to find patterns of associations of sociodemographic characteristics with domains of eHealth usage in the population of US patients with chronic conditions who have access to their online records. The use of online records to securely message health care providers in the past 12 months and the use of online records to request prescription refills in the past 12 months were analyzed using Poisson regression with a log link and robust estimates of standard errors [39]. The use of online records to make decisions on how to treat an illness or condition in the past 12 months and the use of online records to securely share health records with another provider in the past 12 months were analyzed using logistic regression. To account for the complex survey design used to collect the data, we used jackknife replicate weights to compute accurate standard errors, with all analyses weighted to provide nationally representative estimates. We conducted all statistical analyses using SAS 9.4^®^ and Stata 16^®^. The threshold for the significance of the *p*-value was set to ≤0.05.

We summarized the data using appropriate descriptive statistics such as frequency (percent) and weighted percent (standard error). We presented the multivariable regression models using incident rate ratios (IRR) and odds ratio (OR) with a 95% confidence interval (CI).

## 3. Results

### 3.1. Descriptive Results

The final analytic sample comprising respondents with at least one chronic condition that accessed their online medical record at least once in the past 12 months resulted in 1552 participants from the two HINTS cycles. We have presented the summary of the sociodemographic characteristics of the respondents in Table 1. The analytic sample was 56% female, 42% 18–49 years old, 70% non-Hispanic White, 78.2% with some college education, 58% employed, and 47.2% with an income of $75,000 or more. We combined Asian and other races for the purpose of the multivariable analysis. The sample analyzed was 88.8% US born, and 93% reported speaking English very well. Additionally, 96.4% had access to health insurance, 83% had a regular health care provider, and over 73% of the participants reported having a family history of cancer.

Table 2 presents the clinical characteristics of the respondents. About 59% of the analytic sample were never smokers. Overall estimates suggest that about 56% of respondents reported having more than one chronic condition. Within the analytic sample, the most prevalent chronic condition was high blood pressure, with 55% of the respondents reporting having high blood pressure. However, about 45% reported being in very good or excellent health, and less than 20% reported being in fair or poor health.

We have presented the summary for the outcome variables in Table 3. Of all the respondents who accessed their medical records in the past 12 months, 47.3% of the participants used it 1–2 times, while less than 10% accessed their medical record 10 or more times. In the EHR communication domains, 48% of the respondents used the online medical record system to securely message their health care provider, 43.8% of the participants used the online medical record system to request a refill of medications, 21.1% of the respondents used their online medical record system to make a decision on how to treat an illness or condition, and 12% securely shared their medical record with another provider.

### 3.2. Multivariable Model

We have presented the results of the multivariable model in Table 4. Among HINTS respondents with chronic conditions who accessed their online medical record at least once in the past 12 months, respondents 65 to 74 years and those 75 years or older were significantly less likely to use the system to securely message their health care providers compared to respondents 18 to 34 years (IRR = 0.73, 95% CI = 0.57, 0.94; and IRR = 0.54, 95% CI = 0.35, 0.83) respectively.

The results of the multivariable model indicate that race, age, and survey years are associated with respondents who reported the use of the online medical record to make a decision about treating a condition or an illness. Respondents in the 65 to 74 age range had reduced odds of using the medical record to make a decision regarding treating a condition or illness (OR = 0.30, 95% CI = 0.11, 0.84). There was a significant increase in the use of the online medical record to make a decision on how to treat a condition or illness from HINTS 5 cycle 1 to HINTS 5 cycle 2 (OR = 1.99, 95% CI = 1.23, 3.20). Additionally, respondents in the other racial category had increased odds of using the online medical record to make decisions on how to treat a condition or illness in the past 12 months (OR = 2.64, 95% CI = 1.12, 6.24).

Those in the 75 years and older age range had reduced odds of securely sharing their online record with another provider compared to respondents aged 18 to 34 years (OR = 0.17, 95% CI = 0.03, 0.99). Respondents with multimorbidity (2 or more diagnoses) had significantly increased odds of securely sharing their online record with another provider in the past 12 months (OR = 2.04, 95% CI = 1.16, 3.59). Lastly, respondents in the other racial category had increased odds of securely sharing their online medical record with another provider in the past 12 months compared to Non-Hispanic Whites (OR = 3.61, 95% CI = 1.25, 10.42).

The sociodemographic characteristics of the respondents were not associated with their likelihood of requesting prescription refills using the online medical record. No other predictors of interest were significantly associated with any of the outcomes after adjustment for covariates.

## 4. Discussion

To our knowledge, this is the first study to examine disparities in accessing patient portals for disease management among chronically ill noninstitutionalized adults using nationally representative data. Our study examined a broad range of reasons for the use patient portals for disease management, including secure messaging with providers, requesting medications refills, and sharing medical records with other providers.

Our study is consistent with previous studies examining eHealth communication in US adults, revealing that age disparities exist in the use of eHealth communication methods, with older participants having a lower rate of eHealth communication across multiple domains [26,27,29,40]. However, our study yielded specific insights into the use of the patient portals, while the other studies were focused on the more general use of the eHealth domain of communication, such as personal email, searching for health information, buying medications online, and sharing health content though social networking sites [26,27,29,40]. Other studies of seniors 65–79 years old revealed that patients aged 70 years and older were less likely to register to use web portals [24,25]. Some potential explanations as to why older respondents were less likely to be engaged in use of the patient portals include the accessibility of the patient portals, safety concerns, and the usefulness of the portal for communication as opposed to face-to-face meetings with providers. This result, however, is concerning, as the likelihood of using the patient portal may be reflective of the ability to use telehealth services, and because the COVID-19 pandemic has accelerated the expansion of telehealth services [41]. Since a recent study of older adults’ readiness to engage in eHealth and mHealth indicated that over 80% of the respondents reported having access to the internet at home, and 44% of those using the internet reported doing so on a mobile device [42], further studies should be done to understand the digital divide for older patients and how to engage them in eHealth.

In a prior HINTS study of HRIU use among cancer survivors, there has been an increasing trend in the use of HRIU [21,43]. In our study, we similarly found an increase in the use of EHR to make clinical decisions about how to treat an illness or condition. However, no increasing trend was noted in any of the other domains analyzed. This result underscores the importance of encouraging and promoting the use of the portal for other aspects of chronic disease management, particularly communicating with providers and sharing medical records with other providers. Despite the increasing trend in HRIU among cancer survivors, in a prior HINTS study of cancer survivors, there were age, race, education, and geographic disparities in the use of HRIU [27,44]. This study is in line with our result of lower use of online medical records for secure communication, to treat medical conditions, and to share with other providers among older participants. With the recent COVID-19 pandemic, health care providers have shifted to the use of telehealth for primary and specialty care for disease management [45]. The shift to telehealth during the pandemic could further widen the gap of health outcomes for patients with low and limited access to technology and those who are not ready to adopt new or emerging technologies.

Our study indicates that respondents with two or more chronic conditions were 2.04 times more likely to share the EHR with another provider. This result may be attributed to the need for different specialists to be involved in the care of patients with multimorbidity. This current finding is somewhat in line with other study that indicated that patients with chronic conditions were more engaged in care-seeking behavior based on health-related internet searches [36]. Patients with multimorbidity should be encouraged to participate in other forms of eHealth communication through the patient portal for disease management.

Prior studies have found sociodemographic disparities in the use of HRIU [23,28,29,46]. In our study, those identified as other race (Asian and others) were significantly more likely than those identified as White to use the portal for clinical decision making and transferring medical records to another provider. However, only 3.63% of the participants self-identified as Asian, and only 2.95% identified as other, limiting any form of generalization of the results. Although sociodemographic disparities exist in who is being offered access to EHR [47], in our study, which focused on only respondents who reported accessing their EHR at least once in the preceding 12 months, characteristics such as income and education level were not associated with the different domain of using EHR. We suspect that the result is due to our restriction of the analytic sample to respondents who reported having accessed the web portal at least once in the past 12 months. This subset of respondents is important because those respondents were not only offered access to their EHR, but have indicated using it. This restriction on the inclusion criteria for the final analytic sample could potentially control for some of the disparities that exist in accessing EHR, such as access to and use of the internet [26], and being offered its use [47]. For that reason, we did not adjust for the access/use of internet because using the web portal is an indication of internet access/usage.

## 5. Study Strengths and Limitations

There are several strengths to this study. First, the HINTS survey is a national survey of adults in the United States 18 and older. Thus, the subsample used in this study represents adults in the United States living with chronic conditions who have accessed their online health portal at least once in the past 12 months. We also used two HINTS cycles, allowing us to see changes in the use of EHR over time. Additionally, the study included an exploration of different reasons for using the EHR in disease management.

Despites the strengths, our study has some limitations. Given that HINTS is a cross-sectional study, we cannot make conclusions about causality. Furthermore, the survey has participants with a limited number of chronic conditions. Therefore, we can assess the association of respondents’ characteristics with reasons for using EHR only in a subset of patients with chronic conditions. Future iterations of the survey should ask participants about other prevalent chronic conditions. In addition, future iterations of HINTS should include questions that address barriers to using the online medical records.

## 6. Conclusions

A recent HINTS brief indicated that the proportion of US adults accessing their online medical records increased from 27% in 2014 to 40% in 2018 [48]. Despite the increase in the access and use of EHR, our study reveals age as a factor of disparity in assessing the EHR for health care management. However, past studies have shown that over 80% of older adults are already using the internet, and 44% have access to smartphones. Therefore, health care providers should develop strategies to inform older patients and their proxies about the accessibility of the EHR as a secure means of communication to providers. Some strategies can include the development of user-friendly patient portals for multiple platforms that encourage greater use. Other strategies should include eHealth literacy programs that address patients’ concerns regarding safeguarding their protected health information. Additional technological improvements may include the use of integrated displays to decrease user cognitive load [49,50,51], integration of linked EHR records at the household level to facilitate delivery of services, and embedding of health literacy tools (e.g., embedded medical search engines, integrated AI voice chatbots for on-demand self-care advice) to facilitate meaningful patient engagement.

The use of electronic patient portals raises challenges and ethical issues regarding older adults. Notably, disparities in internet access, a key factor for the use of e-health, persist among underserved populations such as older adults and individuals of lower socioeconomic status [52,53]. Older adults may encounter more challenges in the use of EHR and electronic patient portals because older generations must learn and acquire the necessary skills needed to navigate the internet and are less comfortable using technology compared to their counterparts. Furthermore, some older adults with certain illnesses may require support to navigate the complexities of eHealth portals [54]. Our findings are consistent with prior work using HINTS data, which showed that older US adults were less likely to engage in eHealth, and point to a need for additional support to ensure equitable access to e-health for older adults [22]. Other ethical aspects that are of importance are autonomy, privacy, confidentiality, consent, and beneficence [55]. Those ethical issues can be extended to the adaptability and accessibility of patient portals for personal use by patients. With older participants being less likely to engage in eHealth communication through the patient portal, adopters of EHR should consider the issue of autonomy, privacy, confidentiality, and equality of access as they encourage older patients and their proxies to make full use of the system for disease management.

## Figures and Tables

**Table 1:** Sociodemographic characteristics of HINTS respondents with at least one chronic condition who accessed their patient portal at least once in the past 12 months (*N* = 1552).

Sociodemographic Characteristics	*n*	Unweighted Percent	Weighted Percent (SE)
Age			
18–34	120	7.73	14.53 (1.88)
35–49	301	19.39	28.20 (2.01)
50–64	579	37.31	35.10 (1.78)
65–74	376	24.23	13.91 (0.84)
≥75	147	9.47	6.98 (0.72)
Missing	29	1.87	1.27 (0.29)
Gender			
Male	554	35.70	40.46 (1.79)
Female	917	59.09	55.59 (1.80)
Missing	81	5.22	3.95 (0.71)
Race/Ethnicity			
Non-Hispanic White	1036	66.75	70.13 (1.59)
Non-Hispanic Black	178	11.47	7.92 (0.77)
Hispanic	136	8.76	11.48 (1.24)
Asian	51	3.29	3.63 (0.77)
Other	60	3.87	2.95 (0.57)
Missing	91	5.86	3.88 (0.65)
Education			
High school or Less	226	14.56	20.64 (1.58)
Some College or More	1307	84.21	78.24 (1.61)
Missing	19	1.22	1.12 (0.39)
Employment			
Employed	811	52.26	58.18 (1.92)
Unemployed	722	46.52	41.12 (1.93)
Missing	19	1.22	0.71 (0.21)
Income			
Less than $20,000	131	8.44	8.53 (1.11)
$20,000 to <$35,000	144	9.28	8.05 (1.07)
$35,000 to <$50,000	174	11.21	11.51 (1.28)
$50,000 to <$75,000	296	19.07	18.23 (1.43)
$75,000 or More	682	43.94	47.19 (1.98)
Missing	125	8.05	6.49 (0.74)
Born in the United States			
Yes	1373	88.47	88.81 (1.25)
No	158	10.18	10.41 (1.27)
Missing	21	1.35	0.78 (0.22)
Health Insurance			
Yes	1512	97.42	96.35 (1.01)
No	30	1.93	3.34 (1.01)
Missing	10	0.64	0.31 (0.13)
Regular Provider			
Yes	1325	85.37	82.89 (1.56)
No	212	13.66	15.81 (1.53)
Missing	15	0.97	1.30 (0.54)
Family History of Cancer			
Yes	1174	75.64	73.89 (1.91)
No	348	22.42	24.76 (1.88)
Missing	30	1.93	1.35 (0.32)
HINTS 5 Survey			
Cycle 1	736	47.42	47.95 (1.92)
Cycle 2	816	52.58	52.05 (1.92)

SE: Standard Error.

**Table 2:** The Clinical characteristics of HINTS respondents with at least one chronic condition who accessed their patient portal at least once in the past 12 months (*N* = 1552).

Clinical Characteristics	*n*	Unweighted Percent	Weighted Percent (SE)
General Health			
Excellent	132	8.51	8.12 (0.89)
Very Good	607	39.11	37.13 (1.97)
Good	557	35.89	39.09 (1.98)
Fair	207	13.34	13.46 (1.18)
Poor	40	2.58	2.62 (0.57)
Missing	9	0.58	0.57 (0.22)
Smoking Status			
Current	152	9.79	11.67 (1.21)
Former	471	30.35	29.45 (1.52)
Never	912	58.76	57.90 (1.94)
Diabetes			
Yes	423	27.26	24.70 (1.52)
No	1119	72.10	74.66 (1.53)
Missing	10	0.64	0.65 (0.29)
High Blood Pressure			
Yes	884	56.96	54.87 (1.95)
No	654	42.14	44.45 (1.99)
Missing	14	0.90	0.68 (0.25)
Lung Disease			
Yes	272	17.53	15.95 (1.30)
No	1273	82.02	83.76 (1.29)
Missing	7	0.45	0.29 (0.13)
Heart Conditions			
Yes	195	12.56	11.26 (1.37)
No	1349	86.92	88.43 (1.37)
Missing	8	0.52	0.31 (0.12)
Depression			
Yes	547	35.24	39.88 (1.76)
No	997	64.24	59.87 (1.76)
Missing	8	0.52	0.25 (0.11)
Arthritis			
Yes	621	40.01	33.84 (1.45)
No	925	59.60	65.83 (1.46)
Missing	6	0.39	0.33 (0.19)
Cancer			
Yes	357	23.00	14.96 (1.08)
No	1192	76.80	84.98 (1.09)
Missing	3	0.19	0.05 (0.03)
Multimorbidity			
Yes	958	61.73	56.03 (1.72)
No	594	38.27	43.97 (1.72)

SE: Standard Error.

**Table 3:** Summary statistics of patient portal-related communication (*N* = 1552).

Use of Online Medical Record	*n*	Unweighted Percent	Weighted Percent (SE)
Number of times you accessed your record online in the past 12 months?			
1–2 times	695	44.78	47.27 (1.92)
3–5 times	523	33.70	32.94 (1.90)
6–9 times	172	11.08	10.09 (0.94)
10 times or more	162	10.44	9.70 (1.05)
Used online record to securely message a health care provider in the past 12 months			
Yes	750	48.32	50.05 (2.03)
No	742	47.81	46.47 (2.04)
Missing	60	3.87	3.48 (0.63)
Used online record to request a refill of medications in the past 12 months			
Yes	680	43.81	42.07 (1.97)
No	820	52.84	54.75 (1.96)
Missing	52	3.35	3.17 (0.61)
Used online record to make a decision on how to treat illness or condition in the past 12 months			
Yes	327	21.07	20.81 (1.62)
No	1170	75.39	75.89 (1.70)
Missing	55	3.54	3.30 (0.61)
Used online record to securely share it with another provider in the past 12 months			
Yes	186	11.98	12.02 (1.15)
No	1314	84.66	84.79 (1.21)
Missing	52	3.35	3.19 (0.62)

SE: Standard Error.

**Table 4:** Multivariable Model for Patient Portal-related Communication (*N* = 1552).

Sociodemographic Characteristics	Securely Message Provider in the Past 12 Months		Request PrescriptionRefills in the Past 12 Months		Make a Decision on How to Treat Condition in the Past 12 Months		Securely Share it with Other Providers in the Past 12 Months	
	IRR	95% CI	IRR	95% CI	OR	95% CI	OR	95% CI
Race/Ethnicity								
Non-Hispanic White	1.00	1.00, 1.00	1.00	1.00, 1.00	1.00	1.00, 1.00	1.00	1.00, 1.00
Non-Hispanic Black	0.94	0.71, 1.25	1.11	0.84, 1.49	1.46	0.77, 2.79	1.85	0.84, 4.07
Hispanic	0.84	0.62, 1.13	0.99	0.71, 1.37	1.08	0.41, 2.88	0.79	0.25, 2.45
Other	1.09	0.79, 1.50	1.10	0.75, 1.60	2.64	1.12, 6.24 *	3.61	1.25, 10.42 *
Age								
18–34	1.00	1.00, 1.00	1.00	1.00, 1.00	1.00	1.00, 1.00	1.00	1.00, 1.00
35–49	0.98	0.77, 1.26	1.08	0.68, 1.72	0.93	0.37, 2.35	0.80	0.30, 2.13
50–64	0.77	0.58, 1.01	1.08	0.70, 1.68	0.49	0.19, 1.25	0.48	0.18, 1.28
65–74	0.73	0.57, 0.94 *	1.06	0.64, 1.74	0.30	0.11, 0.84 *	0.34	0.09, 1.22
≥ 75	0.54	0.35, 0.83 **	1.14	0.65, 2.01	0.70	0.22, 2.16	0.17	0.03, 0.99 *
Gender								
Male	1.00	1.00, 1.00	1.00	1.00, 1.00	1.00	1.00, 1.00	1.00	1.00, 1.00
Female	0.99	0.83, 1.18	0.83	0.68, 1.02	1.02	0.61, 1.71	0.99	0.55, 1.79
Education								
High School or Less	0.87	0.67, 1.14	0.87	0.64, 1.19	1.03	0.47, 2.27	0.67	0.26, 1.73
Some College	1.00	0.82, 1.21	0.95	0.75, 1.22	0.91	0.50, 1.65	1.02	0.50, 2.07
College Graduate or More	1.00	1.00, 1.00	1.00	1.00, 1.00	1.00	1.00, 1.00	1.00	1.00, 1.00
Income								
Less than $20,000	0.87	0.59, 1.29	0.73	0.43, 1.25	1.14	0.39, 3.31	0.37	0.12, 1.18
$20,000 to <$35,000	0.75	0.52, 1.10	1.14	0.76, 1.71	0.85	0.34, 2.12	0.72	0.27, 1.93
$35,000 to <$50,000	0.62	0.44, 0.86 **	1.00	0.73, 1.35	1.56	0.69, 3.53	1.39	0.45, 4.35
$50,000 to <$75,000	0.99	0.78, 1.26	0.98	0.74, 1.31	1.03	0.51, 2.06	0.79	0.36, 1.73
$75,000 or More	1.00	1.00, 1.00	1.00	1.00, 1.00	1.00	1.00, 1.00	1.00	1.00, 1.00
Born in the United States								
Yes	1.00	1.00, 1.00	1.00	1.00, 1.00	1.00	1.00, 1.00	1.00	1.00, 1.00
No	0.92	0.68, 1.23	0.74	0.50, 1.08	0.59	0.27, 1.28	0.81	0.30, 2.16
Multimorbidity								
Yes	0.93	0.78, 1.11	1.20	0.97, 1.48	1.48	0.86, 2.57	2.04	1.16, 3.59 *
No	1.00	1.00, 1.00	1.00	1.00, 1.00	1.00	1.00, 1.00	1.00	1.00, 1.00
HINTS 5 Survey								
Cycle 1	1.00	1.00, 1.00	1.00	1.00, 1.00	1.00	1.00, 1.00	1.00	1.00, 1.00
Cycle 2	1.17	1.00, 1.36 *	1.07	0.88, 1.30	1.99	1.23, 3.20 **	1.28	0.77, 2.11

The multivariable regression model (logistic and Poisson) was adjusted for insurance status, employment status, having a regular provider, general health status, smoking status, and family history of cancer. We present the results of the Poisson regression models as incident rate ratios (IRR) and the results of the logistic regression models as odds ratio (OR) with 95% confidence interval (CI). * *p*-value ≤ 0.05, ** *p*-value ≤ 0.01.

## Data Availability

The data that support the findings of this study are available from https://hints.cancer.gov/data/download-data.aspx.
